# Water Extract of *Pulsatilla koreana* Nakai Inhibits Osteoclast Differentiation and Alleviates Ovariectomy-Induced Bone Loss

**DOI:** 10.3390/ijms252111616

**Published:** 2024-10-29

**Authors:** Dong Ryun Gu, Hyun Yang, Seong Cheol Kim, Sung-Ju Lee, Hyunil Ha

**Affiliations:** KM Convergence Research Division, Korea Institute of Oriental Medicine, Yuseong-daero 1672, Yuseong-gu, Daejeon 34054, Republic of Korea; mrwonsin@kiom.re.kr (D.R.G.); hyunyang@kiom.re.kr (H.Y.); cheol0907@kiom.re.kr (S.C.K.); sungjulee@kiom.re.kr (S.-J.L.)

**Keywords:** *Pulsatilla koreana* Nakai, osteoporosis, ovariectomy, osteoclast

## Abstract

*Pulsatilla koreana* Nakai (*P. koreana*) is a perennial herb traditionally used to treat malaria and fever. Although the pharmacological properties of *P. koreana* have been explored in various contexts, its effects on bone diseases, such as osteoporosis, remain poorly studied. In this study, we investigated the effects of water extracts of *P. koreana* (WEPK) on osteoclasts, which play a crucial role in bone remodeling, and an ovariectomized (OVX) mouse model, which mimics osteoporosis. Phytochemical profiling of WEPK revealed several compounds that regulate bone or fat metabolism. WEPK suppressed osteoclast differentiation by downregulating the expression of receptor activator of nuclear factor-κB ligand (RANKL), a cytokine that induces osteoclastogenesis. Additionally, WEPK directly inhibited RANKL-induced differentiation of osteoclast precursors by downregulating nuclear factor of activated T cells 1 (NFATc1), the master transcription factor for osteoclastogenesis, by modulating its upstream regulators. In vivo, oral administration of WEPK suppressed bone loss, reduced weight gain, and mitigated fat accumulation in the liver and gonadal tissues of OVX mice. Given its positive impact on bone and fat accumulation under estrogen deficiency, WEPK may serve as a promising alternative therapy for postmenopausal osteoporosis, especially when accompanied by other metabolic disorders, such as obesity and fatty liver.

## 1. Introduction

Bone is a highly mineralized and vital organ that supports the body and protects other tissues [1]. It undergoes continuous remodeling, a process involving bone resorption and formation that allows the skeletal system to adapt to physiological demands. This process is primarily governed by two key cell types: bone-resorbing osteoclasts and bone-forming osteoblasts. Osteoclasts are unique, multinucleated cells derived from the monocyte/macrophage lineage, and their differentiation is regulated by various cytokines, including macrophage colony-stimulating factor (M-CSF) and receptor activator of nuclear factor-κB ligand (RANKL). RANKL plays a pivotal role in bone resorption by stimulating osteoclast differentiation and function [2,3,4]. In contrast, osteoblasts arise from mesenchymal stem cells and, upon embedding within the bone, differentiate into osteocytes. Osteocytes express more RANKL than osteoblasts, and they also express osteoprotegerin (OPG), a RANKL decoy receptor, thus playing a crucial role in modulating bone remodeling [5,6]. A delicate balance between resorption and formation ensures the maintenance of bone strength and mineral homeostasis. However, disruptions in this process can lead to pathological conditions such as osteoporosis, characterized by reduced bone mass and structural deterioration [4,7].

Osteoporosis, the most widespread bone disease worldwide, has emerged as a significant public health concern [8,9]. It is characterized by diminished bone mineral density (BMD) and an increased risk of fractures, owing to excessive bone resorption. Osteoporotic fractures are associated with significant morbidity, disability, and healthcare costs, and in severe cases, they may result in fatal complications. Despite advances in therapeutic strategies, challenges remain in managing osteoporosis, particularly owing to its frequent coexistence with other metabolic disorders such as obesity, steatotic liver disease, and dyslipidemia. Given that there may be common pathogenic mechanisms among these metabolic diseases, osteoporosis treatments could have negative effects on these conditions [10]. Consequently, there is an ongoing need to develop diverse and effective treatment options.

In response to these challenges, natural products have attracted increasing attention as alternative therapies for osteoporosis. Natural compounds are often associated with fewer side effects than synthetic drugs and typically target multiple pathways, which may offer broader therapeutic benefits [11]. Natural substances such as genistein, berberine, and oleanolic acid acetate have shown promise in promoting bone health by suppressing osteoclast activity or enhancing osteoblast function [11,12]. *Pulsatilla koreana* Nakai (*P. koreana*), a perennial herb native to Korea, has traditionally been used for its medicinal properties in the treatment of various ailments, including amoebic dysentery, malaria, and fever [13]. Previous studies have demonstrated that extracts of *P. koreana* exhibit antioxidant [14], anti-inflammatory [15], antitumor [16,17], and renoprotective [18] effects. Despite several natural compounds in *P. koreana* extracts, such as hederagenin and oleanolic acid [19], which are beneficial for bone health [20,21], the potential effects of these extracts on bone metabolism and diseases have not yet been explored. Therefore, we investigated the effects of water extract of *P. koreana* roots (WEPK) on osteoclast differentiation and evaluated its protective role in an estrogen-deficiency-induced osteoporosis mouse model.

## 2. Results

### 2.1. Phytochemical Constituents of WEPK

**Figure 1:**
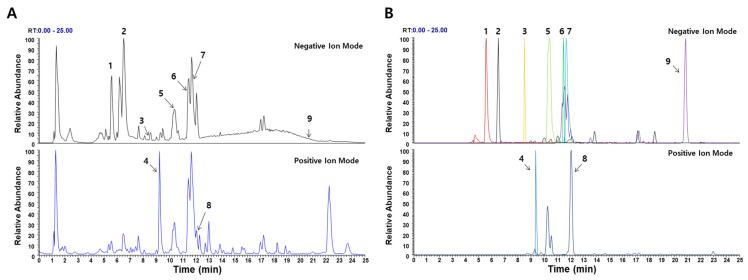
**UHPLC–MS/MS analysis of WEPK:** (**A**) base peak chromatogram; (**B**) extracted ion chromatogram of identified phytochemicals; 1, caffeic acid; 2, chicoric acid; 3, ferulic acid; 4, siderin; 5, sieboldianoside A; 6, hederacolchiside E; 7, sieboldianoside B; 8, hederagenin; 9, oleanolic acid.

Ultra−high−performance liquid chromatography mass spectrometry (UHPLC−MS/MS) analysis was performed to identify the bioactive compounds present in WEPK. Nine compounds were identified (Figure 1 and Table 1). These included three phenolic phytochemicals: caffeic acid, chicoric acid, and ferulic acid, along with six triterpenoids: siderin, sieboldianoside A, hederacolchiside E, sieboldianoside B, hederagenin, and oleanolic acid.

### 2.2. WEPK Impairs Osteoclast Differentiation In Vitro

**Figure 2:**
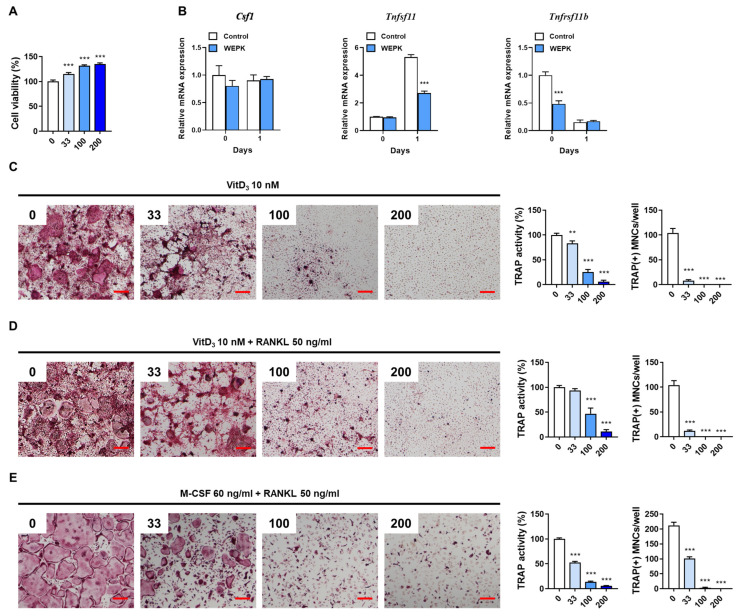
Effects of WEPK on osteoclastogenesis in vitro: (**A**) BMMs were treated with WEPK (0–200 µg/mL) for 1 day, followed by assessment of cell viability. (**B**) MLO-Y4 cells were treated with or without VitD_3_ (10 nM) for 1 day, following pre-treatment with WEPK (200 µg/mL) for 3 h. The mRNA expression of the target genes was assessed by real-time PCR. (**C**,**D**) BMMs and MLO-Y4 cells were cocultured for 5 days with VitD_3_, either without RANKL (**C**) or with RANKL (50 ng/mL, (**D**)), following WEPK pre-treatment. TRAP staining, TRAP activity, and quantification of TRAP-positive osteoclasts (TRAP(+) MNCs) were performed. (**E**) BMMs were cultured for 3 days with M-CSF (60 ng/mL) and RANKL (50 ng/mL) after pre-treatment with WEPK. TRAP staining, TRAP activity, and quantification of TRAP(+) MNCs were performed. Scale bar, 200 μm, ** *p* < 0.01, *** *p* < 0.001 vs. control.

To investigate the impact of WEPK on osteoclastogenesis, we used bone marrow-derived macrophages (BMMs) as osteoclast precursor cells and osteocyte-like cells, MLO-Y4. Initially, we assessed the cytotoxicity of WEPK in BMMs and confirmed that it had no cytotoxic effects (Figure 2A). Osteocytes are known to regulate osteoclast differentiation by expressing osteoclastogenic cytokines, including M-CSF, RANKL, and OPG [5,22]. Therefore, we investigated the effect of WEPK on the expression of these cytokines. Treatment with 1α,25-dihydroxyvitamin D3 (VitD_3_) increased Tnfsf11 (encoding RANKL) expression, decreased Tnfrsf11b (encoding OPG) expression, and did not affect Csf1 (encoding M-CSF) expression in MLO-Y4 cells. In contrast, WEPK inhibited Tnfsf11 expression induced by VitD_3_ without altering Csf1 expression, although it reduced Tnfrsf11b expression in the absence of VitD_3_ (Figure 2B).

Next, we investigated whether WEPK inhibits VitD_3_-induced osteoclastogenesis in a coculture system of MLO-Y4 and BMMs. WEPK dose-dependently decreased tartrate-resistant acid phosphatase (TRAP) activity, a marker of osteoclast differentiation, and also significantly reduced the number of TRAP-stained mature osteoclasts with three or more nuclei and a size greater than 100 μm (Figure 2C). These inhibitory effects were maintained even in the presence of exogenous RANKL, which was added to compensate for the reduced RANKL expression in MLO-Y4 cells after WEPK treatment (Figure 2D). This suggests that the ability of WEPK to inhibit osteoclast differentiation does not solely result from RANKL downregulation. To further investigate whether WEPK directly affects osteoclast precursors, we cultured BMMs without MLO-Y4 cells. WEPK suppressed osteoclast differentiation in the BMM-only culture (Figure 2E). Collectively, these results demonstrate that WEPK not only reduces the osteoclastogenic potential of MLO-Y4 cells but also directly inhibits the differentiation of osteoclast precursor cells.

### 2.3. WEPK Downregulates Osteoclastogenic Transcription Factors and Their Target Genes

**Figure 3:**
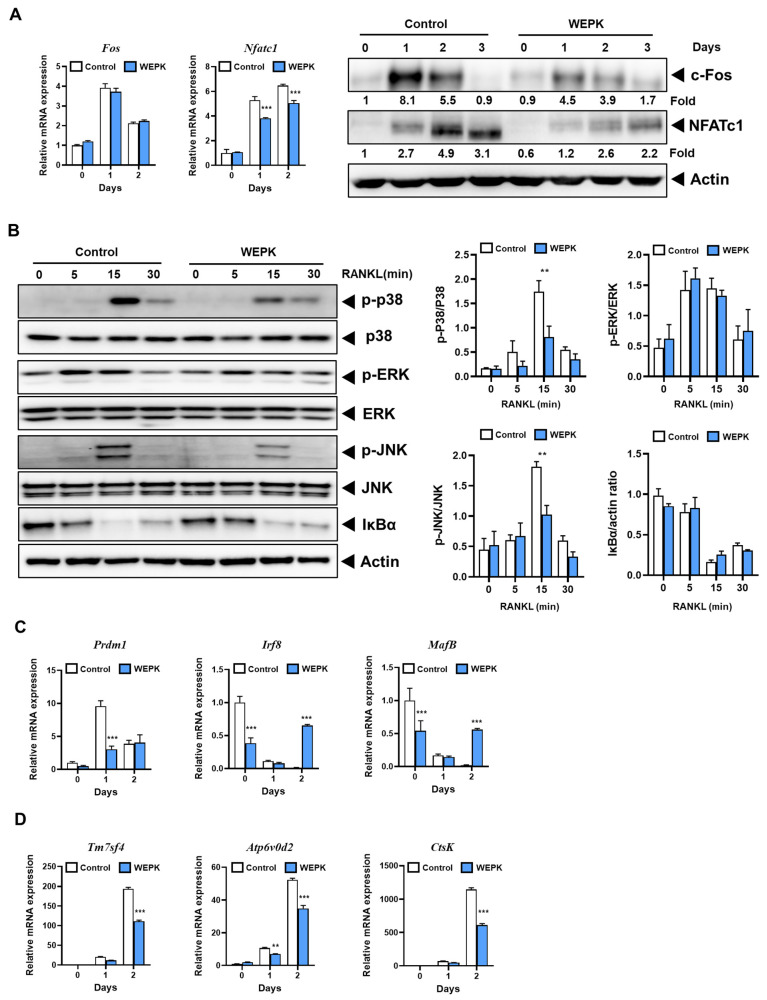
Effects of WEPK on RANKL-induced signaling pathways: BMMs were pre-treated with WEPL (200 µg/mL) and cultured for the indicated times in the presence of M-CSF (60 ng/mL) and RANKL (50 ng/mL). (**A**) The mRNA and protein expression levels of c-Fos and NFATc1 were analyzed by real-time PCR and Western blotting, respectively. (**B**) The activation of MAPKs and IκBα was assessed by Western blotting. (**C**,**D**) Under the same conditions as in (**A**), the mRNA expression levels of osteoclast differentiation regulatory genes (**C**) and osteoclast-specific genes (**D**) were analyzed by real-time PCR. ** *p* < 0.01, *** *p* < 0.001 vs. control.

To further investigate the inhibitory effect of WEPK on osteoclast differentiation in its precursor cells, we analyzed the expression of the key transcription factors c-Fos and the nuclear factor of activated T cells 1 (NFATc1), both of which play critical roles in osteoclastogenesis [23,24]. As shown in Figure 3A, WEPK did not suppress c-Fos mRNA expression but significantly reduced c-Fos protein levels. This suggests that WEPK may influence the translation or degradation process of c-Fos. NFATc1 was reduced by WEPK at both mRNA and protein levels. To elucidate the pathways underlying WEPK’s suppression of these transcription factors, we evaluated the activation of mitogen-activated protein kinases (MAPKs) and NF-κB, key downstream components of RANK signaling [23,25,26,27]. WEPK inhibited the early activation of JNK and p38 without affecting the activation of NF-κB or ERK (Figure 3B). These findings suggest that WEPK suppresses c-Fos and NFATc1 expression by inhibiting p38 and JNK. We also examined the expression of *MafB* and *Irf8*, both of which function as negative regulators of NFATc1 [28,29], and *Prdm1*, which suppresses these negative regulators [30]. WEPK downregulated *Prdm1* expression in the early stages of osteoclast differentiation while significantly increasing *Irf8* and *MafB* expression (Figure 3C). These findings suggest that WEPK suppresses NFATc1 expression by downregulating *Prdm1*, which in turn upregulates *Irf8* and *MafB* expression. Furthermore, we analyzed the expression of NFATc1-regulated osteoclast-specific genes involved in osteoclast fusion and bone resorption. WEPK treatment decreased the expression of *Tm7sf4* and *ATP6v0d2*, which are involved in osteoclast fusion [31,32], and of *Ctsk*, which is a key gene associated with bone resorption [33] (Figure 3D). Overall, these results suggest that WEPK modulates both positive and negative regulators of NFATc1, leading to a reduction in NFATc1 expression and a subsequent decrease in the expression of genes essential for osteoclast differentiation and function.

### 2.4. WEPK Ameliorates Bone Loss

**Figure 4:**
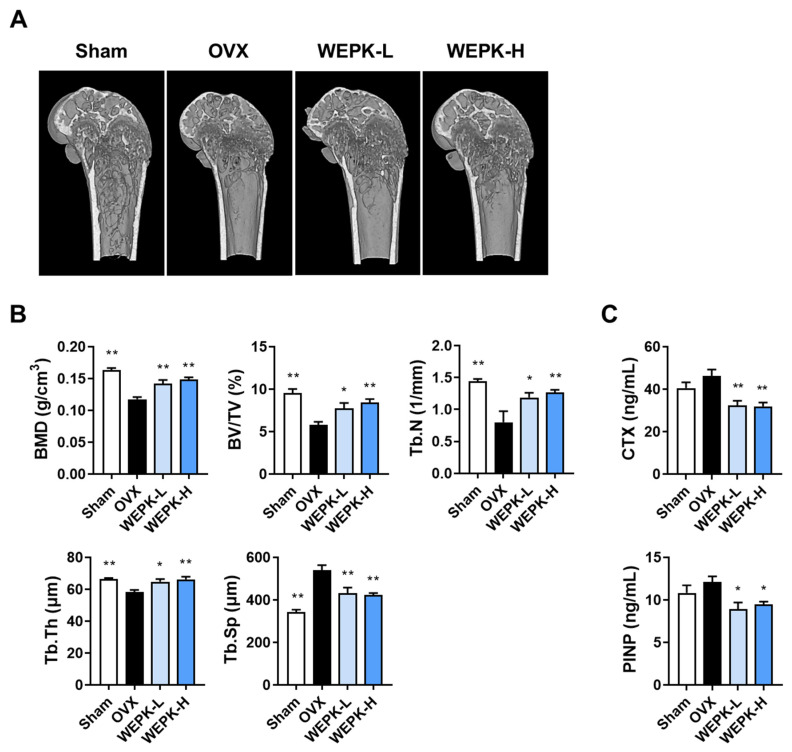
Impacts of WEPK on ovariectomy-induced bone loss: Starting one week after ovariectomy, mice were administered daily at vehicle or WEPK (100 or 300 mg/kg, WEPK-L or WEPKH) for 6 weeks. (**A**) Representative μ-CT images showing the 3D internal structures of the distal femur. (**B**) Quantitative analysis of trabecular bone parameters, including BMD, BV/TV, Tb.N, Tb.Sp, and Tb.Th. (**C**) Measurement of serum levels of CTX and PINP. * *p* < 0.05, ** *p* < 0.01 vs. OVX control. * *p* < 0.05, ** *p* < 0.01 vs. OVX.

To evaluate the potential therapeutic effects of WEPK on osteoporosis, an ovariectomized (OVX) mouse model, a widely used model of osteoporosis [34], was employed. The relationship between the bone and kidney is complex, and the kidney is an organ responsible for regulating the homeostasis of calcium and phosphate, which is essential for bone mineralization [35]. There have been many reports on the physiological and pathological associations between these two organs [35,36]. Based on a previous study in which water extracts of *P. koreana* roots were orally administered at a dosage of 300 mg/kg/day to BALB/c mice to assess renal protective effects [18], we selected dosages of 100 mg/kg/day (WEPK-L) and 300 mg/kg/day (WEPK-H) for these experiments. Following 6 weeks of oral administration of WEPK or vehicle, the distal femora were analyzed by microcomputed tomography (μ-CT). As shown in Figure 4A, the OVX group exhibited significant bone loss compared with the sham group, whereas both the WEPK-L and WEPK-H groups demonstrated a notable reduction in bone loss. BMD, a critical indicator of osteoporosis severity, increased by approximately 20% in the WEPK-L group and by approximately 26% in the WEPK-H group compared to that in the OVX group. Further analysis of bone structure parameters revealed that the bone volume per tissue volume (BV/TV) increased by 33% in the WEPK-L group and 45% in the WEPK-H group. Similarly, the trabecular number (Tb.N) increased by 48% and 58%, respectively. Additionally, a significant increase in trabecular thickness (Tb.Th) was observed, whereas trabecular separation (Tb.Sp) decreased by approximately 20% in both WEPK-treated groups (Figure 4B). Additionally, we measured the serum levels of bone turnover markers, C-terminal cross-linked telopeptides of type I collagen (CTX, indicative of bone resorption), and procollagen type I N-terminal propeptide (PINP, indicative of bone formation). As shown in Figure 4C, both WEPK-treated groups exhibited lower levels of CTX and PINP compared to the OVX group. These changes suggested that WEPK reduces excessive bone resorption in OVX mice while simultaneously affecting bone formation. Collectively, these findings indicated that WEPK exerts a significant anti-osteoporotic effect in the OVX mouse model.

### 2.5. WEPK Alleviates Estrogen Deficiency-Related Fat Accumulation and Liver Injury

**Figure 5:**
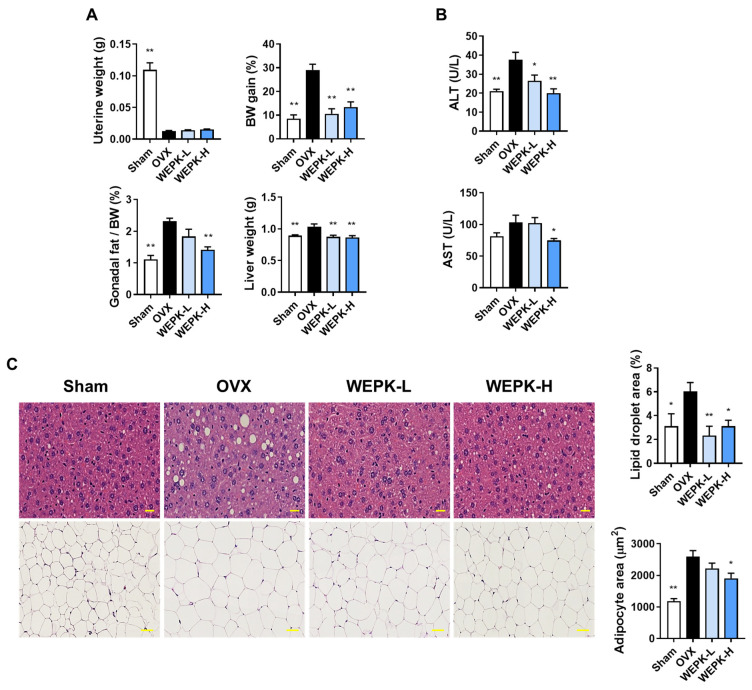
Effects of WEPK on metabolic alterations in OVX mice: Starting one week after ovariectomy, the mice were treated for 6 weeks with either vehicle or WEPK at doses of 100 mg/kg (WEPK-L) or 300 mg/kg (WEPK-H). (**A**) Measurement of uterine weight, body weight (BW) gain, gonadal fat/BW (%), and liver weight. (**B**) Measurement of serum ALT and AST levels. (**C**) Hematoxylin and eosin staining of the liver (top, scale bar 20 μm) and gonadal fat (bottom, scale bar 50 μm). The proportion of lipid droplets in the liver sections and the adipocyte area in the fat tissue images were measured using ImageJ. * *p* < 0.05, ** *p* < 0.01 vs. OVX.

Considering that estrogen deficiency is associated with metabolic alterations, including fat accumulation and liver damage [37,38], we examined the effects of WEPK on these conditions. As shown in Figure 5A, WEPK had no significant effect on uterine atrophy in OVX mice. However, the body weight of OVX mice increased by nearly 30%, whereas WEPK treatment limited the weight gain to less than 20%. In addition, the WEPK-H group exhibited a significant reduction in gonadal fat relative to body weight compared with the OVX group. The liver weights in both WEPK-treated groups remained comparable to those in the sham group. To evaluate the impact of WEPK on liver damage, we measured serum levels of aspartate aminotransferase (AST) and alanine aminotransferase (ALT), both of which serve as indicators of liver injury. ALT levels were lower in both the WEPK-L and WEPK-H groups than in the OVX group, whereas AST levels were significantly reduced in the WEPK-H group (Figure 5B). Further histological analysis of the liver and gonadal fat revealed a marked reduction in liver lipid droplets in both WEPK-treated groups and a decrease in adipocyte size in gonadal fat in the WEPK-H group (Figure 5C). These results indicate that WEPK effectively mitigates fat accumulation and liver damage associated with estrogen deficiency without exhibiting phytoestrogenic effects.

## 3. Discussion

This study revealed various beneficial pharmacological effects of WEPK through in vitro and in vivo experiments. WEPK directly inhibited osteoclast differentiation in BMM, which are the precursors of osteoclasts. Consistent with the in vitro results, WEPK improved bone parameters in the OVX mouse model and reduced serum CTX level, the bone resorption marker CTX. Furthermore, WEPK demonstrated favorable effects on fat metabolism and liver health in OVX mice. Its potential influence on various metabolic disorders suggests that WEPK warrants further investigation.

In the present study, the constituents of WEPK were identified using UHPLC-DAD-MS/MS analysis. Among identified compounds, caffeic acid is associated with bone health, and its derivatives confer beneficial effects on bone integrity [39]. Additionally, both hederagenin and oleanolic acid have been reported to possess bone-protective properties [20,21]. Furthermore, caffeic acid, chicoric acid, and ferulic acid have been investigated for their potential benefits in the context of steatotic liver disease [40,41,42]. The presence of these bioactive compounds in WEPK suggests that they may cooperate or act synergistically to exert beneficial effects on bone and liver; however, further in-depth studies are necessary.

The in vivo findings in the OVX mouse model highlighted the therapeutic potential of WEPK and demonstrated the benefits of natural products with diverse pharmacological effects. WEPK-treated mice exhibited significantly improved bone density and various trabecular bone indicators in the femur, demonstrating the bone-protective effects of WEPK. Furthermore, serum levels of liver injury markers such as ALT and AST were significantly reduced, and histological analyses indicated improvements in fatty liver conditions, indicating the hepatoprotective properties of WEPK. Notably, WEPK also mitigated OVX-induced weight gain and limited the increases in gonadal fat, suggesting its role in fat metabolism. Importantly, WEPK does not exhibit phytoestrogenic effects, thereby reducing potential side effects associated with traditional hormone therapies [43]. Consequently, the development of therapeutics based on WEPK could offer a valuable treatment option not only for bone loss but also for individuals experiencing metabolic disorders resulting from estrogen deficiency.

The mechanisms underlying the anti-osteoporotic effects of WEPK can be inferred from the in vitro results. Previous studies have indicated that *Prdm1* inhibits the expression of negative regulators of NFATc1, such as *Irf8* and *MafB*, during osteoclast differentiation [30]. *Prdm1* conditional knockout mice exhibit increased *Irf8* and *MafB* expression, which suppresses osteoclast differentiation and results in an osteopetrotic phenotype. Consistent with this, WEPK treatment reduced *Prdm1* expression while increasing the expression of *Irf8* and *MafB*, leading to decreased NFATc1 expression and the subsequent inhibition of osteoclast differentiation. Additionally, WEPK inhibited the activation of p38 and JNK, the well-established signaling pathways involved in RANKL-induced osteoclast differentiation [23,25,26], without affecting NF-κB signaling. Given that RANKL-induced activation of MAPKs and NF-κB signaling is dependent on TRAF6 [44], our results imply that WEPK may not disrupt the RANK/TRAF6 interaction. Therefore, through the inhibition of p38 and JNK signaling and the downregulation of *Prdm1*, WEPK suppressed the key transcription factors c-Fos and NFATc1, thereby reducing osteoclast differentiation. These effects on RANKL signaling pathways in osteoclast precursors are likely to significantly contribute to the bone-protective effects of WEPK.

Another mechanism by which WEPK inhibits osteoclast differentiation involves its impact on osteocytes. As key endocrine cells, osteocytes are known to regulate not only bone but also various systemic metabolic processes [45]. In bone remodeling, osteocytes influence osteoblast differentiation by secreting sclerostin and promote osteoclast differentiation by releasing RANKL [46]. While osteoblasts and osteoclasts have relatively short lifespans of days or weeks, osteocytes persist within the bone matrix for considerably longer periods [47]. Thus, targeting osteocytes and their secreted factors is an effective strategy for managing bone diseases [46]. In the present study, WEPK inhibited RANKL expression in MLO-Y4 cells and osteoclast differentiation in cocultures of MLO-Y4 cells and BMMs in response to VitD_3_.

In summary, our study demonstrates that WEPK exerts pharmacological effects by inhibiting osteoclast differentiation and alleviating ovariectomy-induced bone loss, fat accumulation, and liver damage without exerting sex hormone effects. However, further in-depth investigations are essential to elucidate the mechanisms underlying these pharmacological effects, particularly in relation to fat metabolism and hepatic protection. Additionally, the assessment of pharmacokinetic properties and toxicological stability is crucial to validating WEPK as a promising candidate for drug development targeting menopausal metabolic diseases.

## 4. Materials and Methods

### 4.1. WEPK Preparation

WEPK (herb number JW158) was prepared and provided by the National Institute for Korean Medicine Development (Gyeongsan, Korea). After air-drying 0.5 kg of *P. koreana* radixes to remove moisture, the dried radixes were placed in a reflux extractor with 3.5 L of distilled water and extracted for 3 h. The extract was then freeze-dried into a powder, dissolved in distilled water, and filtered through a 0.2 μm filter to obtain WEPK.

### 4.2. Chemical Composition Analysis of WEPK

The chemical composition of WEPK was analyzed using UHPLC-MS/MS according to previously reported methods [48]. The analysis was conducted using a Thermo Dionex UltiMate 3000 HPLC system (Dionex Corp., Sunnyvale, CA, USA) connected to a Thermo Q-Exactive mass spectrometer. Chromatographic separation was performed using an Acquity BEH C18 column (1.7 µm, 100 × 2.1 mm). The mass spectrometer was operated in both the positive and negative ionization modes using a heated electrospray ionization source. Data were acquired and analyzed using TraceFinder software (v 3.2) and Xcalibur software (v 3.0). The chemical components of WEPK were identified by comparing the retention times and mass spectral patterns of the analytes with those of the reference standards purchased from Targetmol (Wellesley Hills, MA, USA).

### 4.3. Preparation of Osteoclast Precursors and Cell Viability Assay

BMMs were prepared from mouse bone marrow cells following the reported method [49]. To measure the cytotoxic effect of WEPK, BMMs were cultured for 24 h in α-minimal essential medium (HyClone, Logan, UT, USA) containing 10% heat-inactivated fetal bovine serum (Thermo Fisher Scientific, Waltham, MA, USA) and 60 ng/mL of M-CSF, treated with vehicle or WEPK (33, 100, and 200 μg/mL). After the treatment, cell viability was assessed by Cell Counting Kit-8 [49].

### 4.4. Osteoclast Differentiation Assay

For in vitro osteoclast differentiation, BMMs and MLO-Y4 were cocultured with VitD_3_, and BMMs were cultured with M-CSF and RANKL, as previously described [49]. The cultured cells were then fixed, and TRAP activity and staining were performed according to established protocols [49].

### 4.5. Quantitative Real-Time PCR

To analyze mRNA expression in MLO-Y4 and BMMs, total RNA was extracted, and cDNA was synthesized from 2 μg of isolated RNA. The cDNA samples were then amplified using TaqMan Universal Master Mix II and the Quant Studio 6 Flex system, employing TaqMan gene expression assays for target genes, including Csf1 (Mm00432686_m1), Prdm1 (Mm00476128_m1), and other genes as previously described [49]. All PCR data were normalized to 18S rRNA as the endogenous control. The relative quantification of target gene expression was determined using the ∆∆Ct method, as described [49].

### 4.6. Western Blotting

Western blotting analysis was conducted as previously described [49]. Briefly, BMMs were lysed using a cell lysis buffer (PRO-PREPTM, iNtRON Biotechnology, Sungnam, Republic of Korea). Equal amounts of protein were subjected to sodium dodecyl sulfate–polyacrylamide gel electrophoresis. The separated proteins were transferred onto polyvinylidene fluoride membranes using a transfer apparatus. Membranes were incubated overnight at 4 °C with a 1:1000 dilution of the primary antibodies, followed by incubation with horseradish peroxidase-conjugated secondary antibodies (1:5000 dilution) at room temperature for 1 h. Antibodies against phospho-p38 (#9211), p38 (#9212), phospho-ERK (#9101), ERK (#9102), phospho-JNK (#9251), JNK (#9252), Ikba (#9242), and Actin (#3700) were purchased from Cell Signaling Technology (Danvers, MA, USA). The other antibodies for NFATc1 (#sc-7294) and c-Fos (#sc-7202), as well as secondary antibodies, were purchased from Santa Cruz Biotechnology (Dallas, TX, USA). The target protein bands were visualized using the ChemiDoc Touch imaging system. The band intensities of NFATc1 and c-Fos were normalized to those of Actin, and the fold changes were calculated.

### 4.7. OVX Mouse Model

Female C57BL/6J (6 weeks old) mice were supplied by SLC Inc. (Shizuoka, Japan) and housed in a specific pathogen-free facility under controlled temperature (22 ± 2 °C), humidity (55 ± 5%), and a 12 h light/dark cycle. The supplied mice were acclimated for one week and then underwent either ovariectomy or sham surgery under anesthesia. Following a one-week recovery period, they were provided with a purified rodent diet containing 10 kcal% fat (D12450B, Research Diets, New Brunswick, NJ, USA). Six mice that underwent sham surgery were administered with vehicle daily (sham group). The remaining OVX mice were allocated to three groups of six mice each: vehicle (OVX group), 100 mg/kg WEPK (WEPK-L group), and 300 mg/kg WEPK (WEPK-H group). WEPK and vehicle were orally administered every day for 6 weeks. After removing the diet from the cages, all mice were anesthetized with avertin (240 mg/kg, i.p.) 7 h later and sacrificed for sample preparation.

### 4.8. Analysis of Bone Microstructure and Quality

The distal femur was scanned using μ-CT (SkyScan 1276, Bruker, Kontich, Belgium) with an X-ray source operating at 85 kV and 47 μA, resulting in a resolution of 2016 × 1344. The images were scanned with a 0.8-degree rotation step and a pixel size of 8 μm. The scanned images were reconstructed into a 3D structure using SkyScan NRecon (version 1.7.42, Bruker). The reconstructed images were analyzed using SkyScan CTAn (version 1.20.3.0, Bruker) to evaluate bone morphometric parameters, including trabecular BMD, BV/TV, Tb.N, Tb.Sp, and Tb.Th. Starting at 80 µm below the growth plate of the femur, 150 cross-sectional images (total length of 1.2 mm) were analyzed.

### 4.9. Measurement of Serum Biomarkers

Blood samples were collected from the caudal vena cava and centrifuged (8000× *g*, 10 min). Serum samples were stored at −80 °C before the measurement of different biomarkers. Serum CTX-I and PINP levels were measured using ELISA kits (Immunodiagnostic Systems Ltd., London, UK). ALT and AST levels were quantified using an automatic biochemical analyzer (Hitachi 7180; Hitachi, Tokyo, Japan).

### 4.10. Histological Analysis

Gonadal fat and liver were briefly washed twice and fixed in 10% formalin at room temperature for two days. Following fixation, tissues were gradually dehydrated using increasing concentrations of ethanol and subsequently embedded in paraffin to create blocks. The paraffin blocks were then sliced into 5 μm thick sections and stained with hematoxylin and eosin. The histologically processed sections were imaged, and the areas of lipid droplets and adipocyte sizes were quantified using ImageJ software (version 1.52a).

### 4.11. Statistical Analysis

We conducted statistical analyses using GraphPad Prism, version 9 (GraphPad, San Diego, CA, USA). The alpha level was set at 0.05 for statistical significance. In vitro results were acquired from three independent experiments and presented as mean ± standard deviation, while in vivo results were derived from six animals per group and presented as mean ± standard error of the mean. To identify significant differences, one-way analysis of variance (ANOVA) followed by Dunnett’s post hoc test or two-way ANOVA followed by Sidak’s post hoc test were used.

## 5. Conclusions

The present study demonstrates that WEPK inhibited osteoclast differentiation by reducing RANKL expression in osteocytes and by blocking RANKL-mediated osteoclastogenesis in osteoclast precursors. Specifically, WEPK downregulated positive regulators of NFATc1, including p38 and JNK, while simultaneously upregulating negative regulators such as *Irf8* and *MafB*, thereby inhibiting RANKL-induced osteoclast differentiation.

Furthermore, using the OVX mouse model, a representative model of menopausal osteoporosis, we demonstrated that WEPK not only provides bone-protective effects but also reduces fat accumulation and alleviates liver damage. These findings suggest that WEPK could serve as a potential candidate for the development of treatments targeting postmenopausal osteoporosis, particularly when accompanied by other metabolic disorders such as obesity and fatty liver.

## Figures and Tables

**Table 1:** Identification of the phytochemicals in WEPK by UHPLC−MS/MS analysis.

No	Retention Time (min)	Ion Mode	Error (ppm)	Formula	Mass (m/z)	MS/MS Fragments (m/z)	Identifications
1	5.56	${\left[M-H\right]}^{-}$	−3.7883	C_9_H_8_O_4_	179.0343	135.0439	Caffeic acid *
2	6.48	${\left[M-H\right]}^{-}$	0.6246	C_22_H_18012_	473.0726	311.0411, 219.0295, 179.0342, 149.0081	Chicoric acid *
3	8.57	${\left[M-H\right]}^{-}$	−2.4572	C_10_H_10_O_4_	193.0502	161.0235, 134.0362	Ferulic acid *
4	9.35	${\left[M+H\right]}^{+}$	−0.160	C_12_H_12_O_4_	221.0808	243.8889, 221.0809, 206.0574, 177.0544, 126.9093	Siderin
5	10.40	${\left[M-H\right]}^{-}$	0.690	C_64_H_104_O_30_	1351.6539	881.4915, 749.4487, 603.3911, 471.3485	Sieboldianoside A
6	11.48	${\left[M-H\right]}^{-}$	0.573	C_65_H_106_O_30_	1365.6696	895.5071, 733.4540, 587.3960, 455.3536	Hederacolchiside E
7	11.71	${\left[M-H\right]}^{-}$	0.634	C_64_H_104_O_29_	1335.6588	865.4964, 733.4544, 587.3963, 455.3535	Sieboldianoside B
8	12.1	${\left[M+H\right]}^{+}$	0.1476	C_30_H_48_O_4_	473.3626	437.3412, 409.3465, 207.1744, 201.1638	Hederagenin *
9	20.82	${\left[M-H\right]}^{-}$	2.5886	C_30_H_46_O_3_	455.3535	-	Oleanolic acid *

*, Compared to the retention time and MS spectral data of the reference standard.

## Data Availability

All relevant data are within the paper.
